# Interplay between Oxidative Stress and Inflammation in Cardiometabolic Syndrome

**DOI:** 10.1155/2016/8254590

**Published:** 2016-08-02

**Authors:** Aaron L. Sverdlov, Gemma A. Figtree, John D. Horowitz, Doan T. M. Ngo

**Affiliations:** ^1^Basil Hetzel Institute, University of Adelaide, Woodville South, SA 5011, Australia; ^2^Boston University School of Medicine, Boston, MA 02118, USA; ^3^Kolling Institute, University of Sydney, St Leonards, NSW 2065, Australia

Around 50–60% of adults in western countries are either overweight or obese with more than 25% falling into the obese category [1]. The metabolic imbalance underlying obesity has fueled the prevalence of cardiometabolic syndrome—a constellation of interrelated risk factors of metabolic origins that together promote the increased risk of cardiovascular disease (CVD) and type II diabetes [2]. These are the major causes of morbidity, mortality, and sky-rocketing healthcare costs in industrialized countries. Obesity is the hallmark component of cardiometabolic syndrome with other key components being insulin resistance, hypertension, dyslipidemia, and endothelial dysfunction. While each of the associated conditions has an independent effect, their clustering has a synergistic effect, making the risk of developing cardiovascular disease greater. Obesity with the associated cardiometabolic syndrome components has a direct effect on atherogenic dyslipidemia, elevated blood pressure, and elevated plasma glucose and promotes proinflammatory and prothrombotic states.

Cardiometabolic syndrome is associated with increased oxidative stress; however, the source of reactive oxygen species (ROS) and their exact targets are not well understood. Oxidation products of different organic molecules including lipids, proteins, and nucleic acids have been used to demonstrate the presence of oxidative stress in patients with cardiometabolic syndrome and CVD. Mitochondria have been shown to be a major source and target of reactive oxygen species in obesity-induced heart disease with consequences being impaired cardiac energetics, development of left ventricular hypertrophy, and diastolic dysfunction [3]. Angiotensin II-induced activation of NADPH oxidase also plays a role, with direct effects via redox posttranslational modifications of proteins within the caveolar compartment. The further downstream effects of ROS and redox regulation are mediated mostly by protein oxidative and nitrosative posttranslational modifications of proteins [4].

Cardiometabolic syndrome has also been associated with the presence of a number of inflammatory markers. Low-grade inflammation is a common manifestation and could play a role in the pathogenesis of obesity and cardiometabolic syndrome and its sequelae. Dysregulation of adipose tissue biology plays a potential role in the initiation of inflammatory events in obesity and cardiometabolic syndrome causing chronic inflammatory response characterized by abnormal adipokine production and the activation of several proinflammatory signaling pathways, resulting in the induction of several proinflammatory cytokines from adipose tissue that have been suggested to play a role in pathogenesis of CVD. Furthermore, the dysregulation adipose tissue biology, mediated by increased redox stress and inflammation, adversely affects angiogenesis both locally and systemically [5], further contributing to the global impact of cardiometabolic syndrome.

It is important to remember that cardiometabolic syndrome frequently coexists with many other diseases, linked by its high prevalence as well as commonalities in aetiology or even causative contribution. A common nexus between many of these disease states is the presence of endothelial dysfunction, mediated either by decreased synthesis and enhanced clearance or by impaired bioaction of nitric oxide.

Rheumatoid arthritis, a common autoimmune inflammatory connective tissue condition, has been associated with increased incidence of CVD. In this issue, T. Dimitroulas et al. investigated the relationship between levels of symmetric dimethylarginine (SDMA), an inactive congener of asymmetrical dimethylarginine, a marker and mediator of nitric oxide synthase inhibition, and inflammatory burden and CVD risk factors in a cohort of rheumatoid disease patients. Interestingly, the authors have not found such an association, potentially related to the interacting effects of antirheumatoid therapy, factors affecting kinetics of SDMA and potentially methodology for SDMA measurement.

Chronic kidney disease is another condition that is intimately linked with incidence of cardiometabolic syndrome and CVD. Kidney disease can result from impaired cardiovascular function but also can in turn lead to worsening cardiac and vascular stiffening and fibrosis. A study by S. Rašić et al., in this issue, suggests that malondialdehyde (an oxidative stress marker) and matrix metalloproteinase-9 (a marker/mediator of fibrosis) are significant predictors of atherosclerosis in chronic kidney disease patients. A. Bakillah et al. looked at the levels of nitrosatively modified proteins in plasma of chronic kidney disease patients undergoing kidney transplantation and found a reduction in nitrated apolipoprotein A-I after transplantation, suggesting reduction in oxidative/nitrosative stress. In an animal model of cardiometabolic syndrome induced in rats by coca cola drinking M. Otero-Losada et al. found induction of cardiac remodeling and renal damage, associated with increase in proinflammatory cytokines, hypertriglyceridemia, and oxidative stress.

While obesity itself has been linked to increased oxidative stress and inflammation, there are many discrepancies in various observational studies. In this issue, S. M. Lee et al. found urinary malondialdehyde and CRP to be positively associated with visceral fat area in a cohort of moderately obese middle-aged men. On the other hand, weight loss has been associated with beneficial cardiometabolic remodeling, but the exact processes that lead to this improvement in metabolic and cardiovascular homeostasis are incompletely understood. S. Karki et al. observed increased expression of adipose tissue lipolytic genes following bariatric weight loss; these correlated with improvements in systemic markers of lipid and glucose metabolism, providing a potential insight into mechanisms of beneficial effects of weight loss.

Obesity and associated sedentary lifestyle are associated with an alteration in a number of secreted metabolic modulators; the precise role of many of these is still poorly understood. Irisin is a hormone secreted from the skeletal muscle under the control of PGC-1*α*, a metabolic master-regulator. Irisin has been suggested to be a mediator of the beneficial effect of exercise, leading to improvement of obesity and glucose homeostasis; however, some controversies regarding its role remain. M. Quiñones et al. found no difference in irisin levels with induction of obesity or manipulation of leptin levels in 2 rodent models, adding to the uncertainties regarding the role and regulation of irisin in obesity.

Poor sleep quality has been linked to development of CVD especially evident in those with disrupted sleep patterns, such as shift workers. T. Kanagasabai and C. I. Ardern investigated the relationship between sleep quality and parameters of oxidative stress and inflammation in a cross-sectional study. They found some mixed results, in general suggesting that fair quality sleepers have the most “optimal” inflammatory and oxidative profile.

Finally, high circulating levels of prothrombotic factors may play an important role in predisposing to prothrombotic episodes, which underlie cardiovascular events seen in obesity. This is further compounded by the increased incidence of atrial fibrillation seen in obese subjects, which in itself confers a much higher thromboembolic risk. N. Procter et al. studied the effect of reduction in left atrial deformation, leading to blood stasis and ultimately thromboembolic events, in atrial fibrillation. The authors did not find a relationship between reduced left atrial deformation and measures of thromboembolic risk, inflammatory activation, or platelet reactivity.

This issue on the interplay of cardiometabolic syndrome, oxidative stress, and inflammation brings together insights from a wide variety of research and disease fields. These studies add to the collective body of knowledge regarding the epidemiology, comorbidities, and mechanistic insights underlying cardiometabolic syndrome and its cardiovascular effects. The overall complexity of the pathophysiology of cardiometabolic syndrome makes these tasks difficult and overwhelming and further studies frequently add more questions than they answer. Developments in redox biomarkers for human application, currently lagging behind their inflammatory counterparts, may assist in dissecting the interaction of oxidative stress and inflammation in a more personalized fashion in the clinic and improve targeted therapeutic strategies. Increased understanding of the mechanistic processes underpinning the development and progression of the cardiometabolic syndrome will allow us to begin to improve outcomes from this syndrome which is a major contributor to mortality, morbidity, and rising healthcare costs in our society.

*Aaron L. Sverdlov* *Aaron L. Sverdlov* *Gemma A. Figtree* *Gemma A. Figtree* *John D. Horowitz* *John D. Horowitz* *Doan T. M. Ngo* *Doan T. M. Ngo*
